# Bilateral Avulsion Fracture of the Fibula Head of the Knee Associated with Avulsion Fracture of the Iliotibial Band: A Rare Case of Fracture Segond Associated with Arcuate Fracture

**DOI:** 10.1155/2020/8825638

**Published:** 2020-07-14

**Authors:** Jonatas Brito de Alencar Neto, Clodoaldo José Duarte de Souza, Márcio Bezerra Gadelha Lopes, Maria Luzete Costa Cavalcante, Luiz Holanda Pinto Neto

**Affiliations:** ^1^Orthopedic Surgeon of the Dr. José Frota Institute, 1816 Barão do Rio Branco St., Fortaleza, Ceará, Brazil; ^2^Orthopedic Surgeon in Articular Clinic Medical Center, 2340 Pontes Vieira Ave., Fortaleza, Ceará, Brazil; ^3^Department of Orthopedics in Walter Cantídio Hospital University Federal of Ceará, 1290 Pastor Samuel Munguba St., Fortaleza, CE, Brazil

## Abstract

Fibular head avulsion fractures are rare and are so-called the arcuate signal. Avulsion fracture of the iliotibial band and anterolateral ligament is known as a Segond fracture, and it is another rare entity. We describe the case of a 27-year-old woman who was hit by a car and suffered polytrauma, mainly suffering injuries to both knees. Radiographs of the knees showed a Segond fracture associated with the arched signal bilaterally. The aim of this study is to present a rare case report and literature review of a bilateral fibular head avulsion fracture associated with an anterolateral tibial avulsion fracture.

## 1. Introduction

Avulsion fractures of the fibular head are rare and are so-called the arcuate signal [1]. The “arcuate signal” is used to describe an avulsed bone fragment related to the insertion site of the tendon of the biceps femoris associated with the arcuate complex, which consists of the fabellofibular, popliteofibular, and arcuate ligaments [2]. Such lesions are typically observed in direct trauma to the knee with excessive varus and internal rotation forces or indirect trauma with the same direction of the force [3]. Avulsion fracture of the iliotibial band (ITB), anterolateral ligament (ALL), and biceps aponeurosis is known as a Segond fracture [4–7]. This fracture is usually associated with acute ACL tears. Helito et al. showed 3/113 Segond fractures in adults and the same authors reported 3/191 in adolescents [8, 9].

The association of fibular head avulsion fracture (arcuate sign) associated with a Segond fracture has not yet been described in the current literature, especially in bilateral cases like this one.

The purpose of this study is to present a case of a 27-year-old woman who was hit by a car and arrived at the Emergency Department presenting polytrauma with an avulsion fracture of the bilateral fibular head associated with avulsion of the ALL complex. The patient underwent surgical fixation of both knees. Rehabilitation was followed by a postoperative physiotherapy protocol leading to fracture consolidation. Despite the severe injury, internal fixation with the repair of the knee's posterior-lateral corner was performed with satisfactory results.

## 2. Case Presentation

A 27-year-old woman who suffered a high-energy trauma after being hit by a car presents herself to the Emergency Department of a trauma hospital level III with multiple trauma. After initial ABDCE by ATLS [10], she was suffering from pain in the pelvis, left arm, and both knees. On physical examination, there was deformity and edema in the left arm with functional disability, but without neurovascular changes. The pelvis presented with pain on palpation, but without instability. The knees were quite edematous without blisters and apparent varus deformity bilaterally. Varus stress was positive grade III [11]. She had a negative anterior and posterior drawer exam. Dorsalis pedis and posterior tibial pulses were normal, as well as the neurological examination of the normal lower limb. The skin was intact for all injuries.

**Figure 1:**
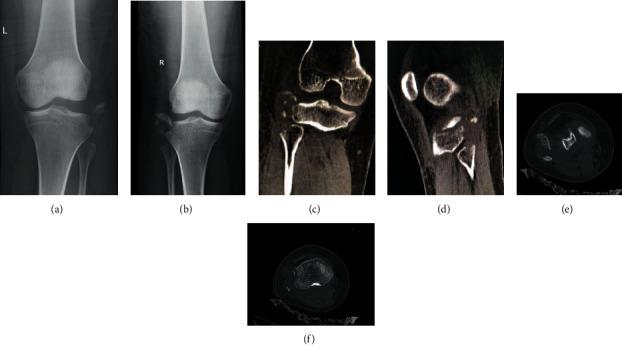
(a, b) Anteroposterior radiography of the knees showing a Segond fracture associated with an arcuate fracture. (c, d) CT of right knee in coronal view and left knee in sagittal view showing avulsion of the fibular head associated with avulsion fracture of the left with small fragments. (e, f) Right knee axial CT sequence.

Left arm radiographic examination showed a distal third left diaphyseal humerus's fracture that was classified as AO/ASIF (Arbeitsgemeinschaft Osteosynthesefragen/Association for the Study of Internal Fixation) 12 B2C [12]. Radiography of the pelvis showed right pubic branch fracture with minimal deviation (Tile/AO A1) [13]. Radiographic examination of the knees showed bilateral avulsion fractures of the fibular head associated with the arcuate signal, being classified as AO/ASIF 41 A19 (Figure 1). For a better definition of the fracture pattern, fragment size, and surgical planning, a CT (Computerized Tomography) scan of both knees was performed (Figure 1). The pelvis fracture was treated nonsurgically. The humerus fracture was initially immobilized, and surgical treatment was performed later with open anatomical reduction and fixation with a dynamic compression plate associated with an interfragmentary screw. Bilateral immobilization on the knees was performed temporarily, and surgical treatment was performed seven days after hospitalization because it was the time needed for edema regression.

The procedure was performed on a radiolucent operating table in a horizontal supine position with spinal anesthesia associated with a regional ultrasound-guided femoral nerve block (LOGIQ S8, GE Healthcare®, USA) with a high-frequency linear transducer (8-15 MHz) [14]. It was performed pneumatic tourniquet with pressure 100 mmHG above the systolic pressure first on the right side. After the first procedure was finished, the pneumatic tourniquet was deflated and the contralateral side surgical procedure was begun. The posterolateral rotatory drawer maneuver and dial test were performed under anesthesia at this time, which were positive lesion of the posterolateral corner [15, 16]. The first knee to be operated (right knee) was placed at 70° of flexion, while the other (left knee) was in full extension on the radiolucent surgical table. A lateral curvilinear hockey stick incision was made parallel to the posterior aspect of the ITB, curving over the lateral femoral condyle towards distally to the area between Gerdy's tubercle and the anterior aspect of the fibular head. An excessively anterior incision in the skin makes access and dissection of the common fibular nerve difficult [17]. Initially, the common fibular nerve was dissected and isolated, leaving it marked with a cardiac tape without any traction.

**Figure 2:**
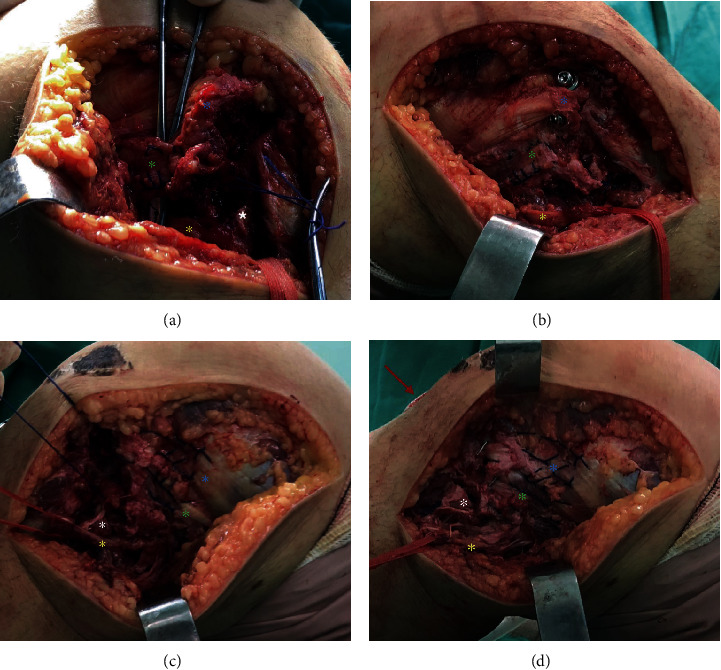
(a) Lateral approach to the right knee showing the tendon of the long head of the biceps repaired by Krackow suture, identified and isolated fibular nerve, fibular head with avulsed fragment and lateral margin of the tibia with the iliotibial band completely inserted to the full. (b) Right knee after Segond fragment fixation and transosseous PLC repair. (c) Lateral approach to the left knee showing extensive injury on the lateral edge of the tibia near the iliotibial band and comminuted avulsion fracture of the fibular head next to the tendon of the long head of the intact biceps. (d) Left knee after Segond fracture fixation with pull out associated with Kirschner wires and transosseous PLC repair (*green asterisk* = tendon of the long head of the biceps; *yellow asterisk* = fibular nerve; *white asterisk* = head of the fibula with avulsed fragment; *blue asterisk* = lateral edge of the tibia with iliotibial band completely inserted in full; *red arrow* = mini access for positioning of pull-out plate).

**Figure 3:**
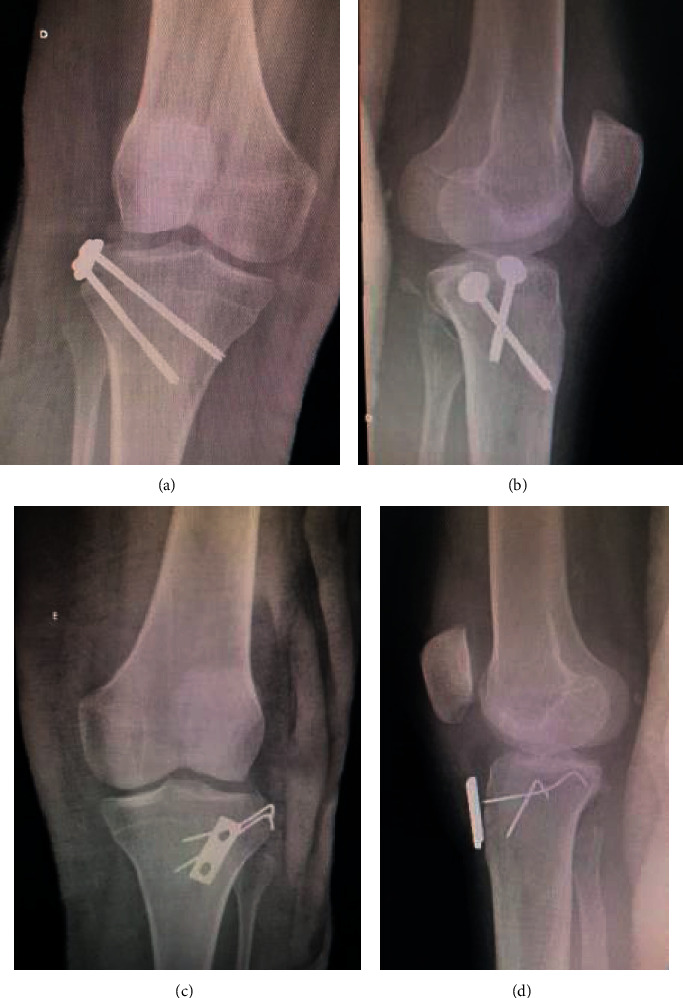
Anteroposterior radiographs pos operative and lateral view of the right knee (a, b) and left knee (c, d).

Fibular head's comminuted fracture with the conjoint tendon (biceps femoris long head and lateral collateral ligament) inserted without injury was identified. Also, it was observed a single large fragment of the lateral edge of the tibia with an ALL complex inserted without injury. Krackow suture was performed with No. 5 ethibond thread in the conjoint tendon together with the posterolateral ligament complex (Figure 2(a)) [18]. The tibial fragment was provisionally fixed with three 2.0 Kirschner wire and, after checking the reduction, definitively fixed with two 4.5 mm cannulated screws and two washers (Medtronic Sofamor Danek Inc., Memphis, TN, USA). Transosseous tunnels were performed in the fibular head (10 mm distal to the fracture line and were 15 mm away from each other) to fix the posterolateral ligament complex (Figure 2(b)). On the left knee, the exact same access was used. Isolation of the fibular nerve and cardiac tape marking was performed. Multifragmentary fracture of the lateral edge of the tibia with the insertion of the integral ALL complex was observed, as well as fibular head avulsion fracture with multiple microfragments with an intact conjoint tendon. Krackow suture was performed in the conjoint tendon together with the posterolateral ligament complex (PLC) and another suture, separately, in the iliotibial band (Figure 2(c)). Transosseous PLC fixation was performed on the fibular head, and the main fragments were fixed on the lateral margin of the tibia with 1.5 Kirschner wires. The ALL complex was fixed by a pull-out technique [19], creating a transtibial tunnel and tied to a 3.5 DCP plate (Medtronic Sofamor Danek Inc., Memphis, TN, USA) in an anterior cortex placed through anterior mini access (Figure 2(d)). Control radiography was performed intraoperatively on both knees (Figure 3).

The postoperative period was performed with a specific physiotherapy protocol similar to that used for PLC ligament injuries [11]. In the immediate postoperative period, the knees were kept in an articulated brace with a range of motion (ROM) gradually allowed (1-2 weeks: 0 -60°; 3-4 weeks: 0-90°). After the 5th week, progression occurred to complete ROM in order to avoid arthrofibrosis. Isometric exercises of quadriceps, hamstrings, sural triceps, and anterior tibialis, as well as active and passive range of motion of the hip, knee, and ankle, were performed from the first postoperative day. In relation to the other lesions, she developed a slight superficial infection of the surgical wound on his left arm in the immediate postoperative period, which was treated only with serial dressings and an oral antibiotic for 5 days. Pubic branch fracture remained well throughout the follow-up, with no deviation. Weightbearing was not allowed in the first four weeks. The brace was discontinued after the first month, being used only during weight-bearing which was started after thirty days of surgery with two axillary crutches. Full weight-bearing was allowed only after two months of surgery. Outpatient returns were performed 15, 30, 60, 120, and 180 days after surgery.

**Figure 4:**
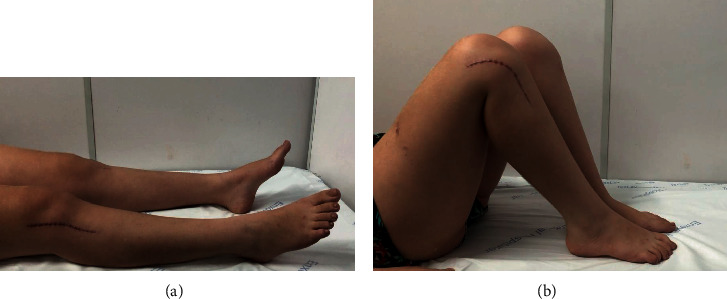
(a, b) Supine photograph showing full range of motion of hips, knees, and ankles.

The patient evolved well in the late postoperative period, showing fracture consolidation after three months of surgery. She had normal walking with a full range of motion of the hip, knee, and ankle (Figure 4). She did not present anteroposterior instability to the stress tests (Lachman, anterior drawer, posterior drawer, and pivot shift), nor valgus and varus instability. She felt no pain after 3 months. She returned to his usual sport (volleyball) after 180 days with a Lysholm functional score 15/95 points (excellent) [20].

## 3. Discussion

Fibular head avulsion fractures are rare. In a retrospective study of 2318 knee injuries, only 13 had suffered this injury (0.6%) [21]. Bilateral traumatic fractures of the fibular head have only been described in two cases: a young woman victim of being hit by a car [1]. The second case was a patient who suffered a seizure which led to an avulsion fracture of both fibular heads due to forced contraction of the femoral biceps [22]. The Segond fracture frequency is uncertain, but it is known that Segond is associated with ACL injuries in 75% of the cases and meniscal injury in 60-70% of the times [3]. Bilateral Segond fracture associated with fibular head avulsion fracture (arcuate sign) has not yet been described in the literature.

The ITB is an important structure in the anterolateral region of the knee. ITB's anatomy was described in 1958 [23]. It includes firm fixations close to the lateral femoral condyle now known as Kaplan fibers. The ALL is another important structure and has received more attention due to the redefinition of the knee's anterolateral anatomy. According to Huang, the “arcuate” sign is used to describe an avulsed bone fragment with a close relation to the insertion of the arcuate complex, which consists of the fabellofibular, popliteofibular, and arcuate ligaments, usually in the styloid process of the fibular head [1, 21]. In that same study, no rupture of the tendon of the biceps femoris muscle was found as well as no evidence of avulsed bone fragment included the origin of the lateral collateral ligament (LCL) nor the biceps femoris tendon. Unlike another study that evaluated 18 patients with an arcuate sign demonstrated insertion of the LCL or biceps tendon in all of them [24]. In our case, the avulsed fragments showed insertion of the tendon of the biceps femoris and the posterolateral ligament complex and these were intact. Biomechanically, ITB acts as a secondary restrictor of anterior tibial translation and a restriction of lateral translation during the pivot shift test in ACL-deficient knees [25]. The posterolateral complex, LCL, popliteal tendon and biceps tendon are the main restrictors of the varus forces and external rotation of the knee [3, 5, 11, 26].

In a study of 13 cases of victims of the Segond fracture, Maeseneer et al. describe the main injury mechanism as varus forces with internal rotation of the knee [4]. In our case, the injury mechanism was similar with an anterior to posterior force on the anteromedial face of the knees, creating varus forces and internal rotation bilaterally associated with hyperextension caused by being run over by a car. This trauma was similar to a case report of bilateral avulsion fracture of the fibular head, but without a Segond fracture [1]. The other case reported in the literature with the same fracture pattern was due to seizure [22]. In neither cases, the patients had other injuries, which differs from the case presented here, which was a polytrauma (there were also a pelvis and a humerus' fractures). There are other lesions associated with a Segond fracture. Medial collateral ligament (MCL) injury, a medial meniscus injury, and a posterolateral corner are some examples [2, 4]. There is also a description of posterior cruciate ligament (PCL) injury [3, 27]. Association with fibular nerve injury can be found in 12-29% of the cases. Vascular injuries are rare [5], but the ABI (Ankle Brachial Index) should always be performed in case of vascular injury suspicion [28]. A prospective study evaluating patients who underwent Magnetic Resonance Imaging (MRI) for acute knee injury demonstrated an overall incidence of 9.1% of PLC injuries and 16% of cases associated with ACL or PCL rupture [17].

In a dissection study of 21 cadavers, the insertion of PLC in the fibular head and its relationship with avulsion fracture (file sign) were evaluated. It was identified that a fracture fragment typically involved a styloid process of the fibular head and had a horizontal orientation, with its size ranging from 8 to 10 mm in length and 2 to 5 mm in width [29]. In a retrospective study of 48 proximal fibular fractures, Cohen et al. analyzed and measured the distance and size of the arcuate fracture fragments. These authors identified a typical arcuate fracture pattern. These patterns are as follows: pattern 1: small avulsions of the fibular styloid; pattern 2: avulsion of a cortical margin of the superior part of the fibular head that surrounds the arcuate complex (classic “arcuate fracture”); and pattern 3: fractures through the metaphyseal bone of the fibular head. The prevalence of these fractures was divided in this study into eleven fractures of pattern 1 (22.9%), six fractures (12.5%) were pattern 2, and thirty-one were pattern 3 (64.58%) [27].

In terms of Segond Fracture treatment, Fay et al. described a case in which there was avulsion of the anterolateral edge of the fibula, ACL injury, LCL injury, and popliteal tendon injury. The authors performed the reconstruction of these structures and fixation of the avulsed fragment, maintaining an immobilizer in the postoperative period [3]. In our case, the Segond fracture was fixed with two compression screws. The fragmented and comminuted side, on the other hand, was fixed with Kirschner wires associated with Krackow repair in the iliotibial band and a pull-out technique in the anterior cortex with a plate. This technique has been described for repair of ACL tibial avulsion [30] or meniscal root injuries [31]. The first case described in the literature of bilateral avulsion fracture of the fibular head was treated nonsurgically without immobilization and with early range of motion allowed, with good results [21]. The other case described, on the other hand, was treated nonsurgically with immobilization by a functional brace, allowing a range of motion, progressing to weight-bearing after the disappearance of pain symptoms and bone bruises [1]. In their report, Harhaji et al. described an isolated case of avulsion fracture of the fibular head with a comminuted fracture pattern which was fixed with a metal wire [2]. In terms of fixation rigidity, in one cadaveric study, the results of the use of anchors in patellar tendon's repair were superior versus transosseous suture [32] which corroborates with another cadaver study for rupture of the quadriceps tendon, where it was found that anchors are also biomechanically superior [33]. A study in 384 shoulders, on the other hand, found that the transbone suture in rotator cuff injuries had a lower cost, better functional results, and a low rate of complications [34]. Zhang et al. described a technique for arched fracture repair with suture using anchors, totally arthroscopic with good results [35]. In our case, the arcuate fractures were fixed with transbone sutures because they are less costly, easier, and with good results described in the literature.

Andrea et al. described a technique according to the fragment size. The suture or anchor fixation was used when the bone fragment was 2 cm^2^ or smaller. There were 3 parallel square knot stitches using the number 2 Vicryl® suture or a 5 mm suture anchor with fixation of the mattress suture while the patient knee was at 90 flexion and neutral rotation. Fragments greater than 2 cm^2^ were fixed by cannulated screws. Plication of the capsule with absorbable suture was performed concomitantly when extensive damage or elongation of the capsule was observed [36].

The postoperative management of avulsion fractures of the lateral margin of the tibia (Segond fracture) is not well described in the literature. All reported cases used knee immobilizers in the postoperative period, varying the length of time used. Weight-bearing and range of motion were quite variable [ 3–5, 26, 28]. In a large study with 27 experienced knee surgeons, it was defined that the brace should be continued for up to 6 weeks. The range of motion was allowed since the first postoperative day. Returning to sport is quite controversial, but it is not recommended before 9 months of knee PLC reconstruction [11]. In our case, we performed articulated brace with a range of motion and isometric exercises allowed since the immediate postoperative period. The return to sport was earlier because we believe that, as it was a bone lesion, the healing time should be faster.

Some limitations must be considered in our study. One of the limitations is the absence of 3D reconstruction CT images. This would help to better delimit the fragments, as well as assist in surgical programming. However, good quality radiographs in addition to the CT compensate this limitation. We did not have access to preoperative or postoperative MRI, making it impossible to visualize associated injuries (ACL, PCL, and meniscus), as well as evaluation of the ITB, tendon of the biceps femoris, and PLC's preoperative integrity. However, we performed intraoperative and late postoperative stress tests where there was no evidence of instability in any plan. The absence of CT for postoperative control is another limitation, which makes it impossible to measure the quality of the reduction accurately, but we have good quality postoperative radiographs, which estimates us an excellent reduction and alignment.

The case was treated in the Brazilian Public Health System, and we had no access to arthroscopic evaluation during the procedure to look for ligament, chondral, nor meniscal injuries.

The fact that it is a single case described is a relevant factor that shows a low level of evidence, but it is a very rare and unique case in the literature, with good documentation, with relevant findings and descriptions of the procedures performed as well as its follow-up.

## 4. Conclusion

This is the first reported case of bilateral Segond fracture associated with an arcuate fracture in the current literature. We believe that the detailed assessment of the integrity of soft parts (tendon of the biceps femoris/PLC and ITB) inserted in the avulsed fragments is one of the main key points in the treatment.

## Data Availability

All the data used in this work are in the possession of the main author (Jonatas Brito) and can be consulted at any time.
